# Interaction effects of visual stimulus speed and contrast on postural sway

**DOI:** 10.1007/s00221-015-4438-y

**Published:** 2015-09-16

**Authors:** Vivian Holten, Maarten J. van der Smagt, Frans A. J. Verstraten, Stella F. Donker

**Affiliations:** Division of Experimental Psychology, Helmholtz Institute, Utrecht University, Heidelberglaan 1, 3584 CS Utrecht, The Netherlands; School of Psychology, The University of Sydney, Griffith Taylor Building A19, Sydney, NSW 2006 Australia

**Keywords:** Postural sway, Vision, Translation, Stimulus contrast, Self-motion

## Abstract

Manipulating the characteristics of visual stimuli that simulate self-motion through the environment can affect the resulting postural sway magnitude. In the present study, we address the question whether varying the contrast and speed of a linear translating dot pattern influences medial–lateral postural sway. In a first experiment, we investigated whether the postural sway magnitude increases with increasing dot speed, as was previously demonstrated for expanding and contracting stimuli. In a second experiment, we also manipulated the contrast of the stimuli. For reasons that high-contrast stimuli can be considered ‘perceptually’ stronger, we expect that higher-contrast stimuli induce more sway than lower-contrast stimuli. The results of the first experiment show that dot speed indeed influences postural sway, although in an unexpected way. For higher speeds, the sway is in the direction of the stimulus motion, yet for lower speeds the sway is in a direction opposite to the stimulus motion. The results of the second experiment show that dot contrast does affect postural sway, but that this depends on the speed of the moving dots. Interestingly, the direction of postural sway induced by a relatively low dot speed (4°/s) depends on dot contrast. Taken together, our results suggest that interactions between the visual, vestibular and proprioceptive system appear to be influenced by an internal representation of the visual stimulus, rather than being influenced by the external visual stimulus characteristics only.

## Introduction

When we move through the environment, our visual, vestibular and proprioceptive system provide coherent information that we are in locomotion. When a stationary observer views a stimulus simulating self-motion through the environment, a sensory conflict between these sensory systems occurs. That is, the visual system provides information that the observer is moving through the environment, yet the proprioceptive system and the vestibular system tell the brain that the observer is stationary. The well-known swinging room experiments of David Lee and colleagues have shown that visual information dominates in this competition between the conflicting sensory inputs, causing the observer to sway in a direction opposite to the direction of the experienced self-motion (e.g., Lishman and Lee 1973; Lee and Aronson 1974; Lee and Lishman 1975).

One could argue that manipulating the characteristics of a visual stimulus simulating self-motion through the environment affects the perceptual strength of this visual stimulus and as a consequence also the behavioral correlate (i.e., postural sway). Several earlier studies have shown that manipulating the visual stimulation indeed influences postural sway (e.g., Flückiger and Baumberger 1988; van Asten et al. 1988a, b; Fushiki et al. 2005). For example, varying the speed of an optic flow pattern simulating self-motion through the environment affects the postural sway magnitude (Lestienne et al. 1977; Wei et al. 2010; Holten et al. 2013). In addition, manipulating the speed gradient of an optic flow pattern (Holten et al. 2013), the motion direction (Lestienne et al. 1977; Palmisano et al. 2009; Wei et al. 2010; Holten et al. 2013) or the spatial frequency (Masson et al. 1995) of a visual stimulus influences postural sway as well.

It is rather difficult to compare the results of studies that investigated postural sway, since the studies used different visual stimuli and different techniques to measure the postural sway. In this study, we will therefore systematically investigate the effect of stimulus contrast and angular velocity on medial–lateral postural sway. Medial–lateral sway has been less studied than anterior–posterior body sway and has one important methodological advantage; several studies have shown that a contracting radial optic flow stimulus induces more postural sway than an expanding stimulus (e.g., Lestienne et al. 1977; Palmisano et al. 2009; Wei et al. 2010; Holten et al. 2013). No such directional anisotropy in medial–lateral sway has been reported for simulated leftward or rightward motion of the observer (e.g., Ehrenfried et al. 2003; Ravaioli et al. 2005; Tsutsumi et al. 2010). Thus, in order to avoid any already known directional anisotropy in postural sway, we present linear translating dots to observers.

The above-mentioned studies have demonstrated that changing the characteristics of a visual stimulus affects postural sway. It is therefore likely that the *perceptual strength*^1^ (e.g., sensitivity to, conspicuity of, bias toward) of a visual stimulus influences postural sway. In general, high-contrast stimuli are probably perceptually stronger visual stimuli than their low-contrast counterparts. For example, the activity in the visual cortex has been demonstrated to increase with increasing stimulus contrast (e.g., Sclar et al. 1990; Boynton et al. 1998; Heeger et al. 2000). Psychophysical studies have shown that increasing the contrast of a visual stimulus leads, for example, to decreased search times in a visual search task (e.g., Näsänen et al. 2001). A high-contrast stimulus breaks suppression earlier than a low-contrast stimulus during continuous flash suppression, indicating a higher conspicuity for the high-contrast stimulus (Tsuchiya and Koch 2005). Given that the above-mentioned previous psychophysical studies have reported that varying stimulus contrast affects behavior (e.g., search times, reaction times), it is possible that postural sway, which is also a behavioral outcome, is affected by a change in stimulus contrast as well. In the present study, we specifically address the question whether systematically manipulating the contrast level of a stimulus simulating self-motion systematically affects the postural sway magnitude. Given the vast amount of evidence, discussed above, we hypothesize that a ‘perceptually stronger’ high-contrast stimulus induces more postural sway than the ‘weaker’ low-contrast version.

Apart from the dot contrast, we also systematically vary the angular velocity of the visual stimulus to examine whether increasing the speed of a linear translating dot pattern increases the magnitude of medial–lateral sway. Previous studies have shown that for stimuli simulating self-motion through the environment in the anterior–posterior direction, the postural sway magnitude increases with increasing stimulus speed (e.g., Lestienne et al. 1977; Wei et al. 2010; Holten et al. 2013), although at the highest speeds, the postural sway magnitude did not increase anymore. Some studies (e.g., Stoffregen 1986) have even reported that for speeds beyond the range of those generated by natural postural instabilities, sway is relatively reduced compared with the postural response induced by lower speeds. For translational motion, the magnitude of medial–lateral sway also appears to depend on stimulus speed. For instance, both Blanks et al. (1996) and Ehrenfried et al. (2003) report an increase in medial–lateral sway with increasing stimulus speed. However, these previous studies used relatively fast translational motion speeds (more than 20°/s). In the current study, we will examine whether a broad range of lower translational dot speeds also causes the sway magnitude to increase with increasing stimulus speed.

All in all, the current study systematically manipulates the angular speed and the dot contrast of a linear translating stimulus. We investigate the effect of each of these parameters on postural sway and expect that perceptually stronger visual stimuli generate more postural sway. If stimulus contrast indeed influences sway, it could be of importance for other studies investigating postural control. Most studies just select a (often arbitrary) stimulus contrast level and measure the effect of another stimulus parameter on sway. However, if contrast were to interact with the other parameter, the contrast level would influence the results. For example, stimulus contrast is known to affect perceived stimulus speed (e.g., Thompson 1982; Thompson et al. 2006), which in turn might affect the sway magnitude. If such an interaction is indeed demonstrated, future studies could take the effect of stimulus contrast on sway into account when analyzing their results.

## Experiment 1

Prior to manipulating the dot contrast of the translating stimulus, we first investigated the effect of the dot speed on the postural sway magnitude. Previous studies have shown that the sway path length (Ehrenfried et al. 2003) and the sway amplitude (Blanks et al. 1996) increase with increasing speed of the translatory stimulus. Both studies have used relatively fast (more than 20°/s) stimulus speeds. In the first experiment of the current study, we examined whether lower translational dot speeds also affect the sway magnitude. Analogous to the findings of previous studies using radial optic flow stimuli simulating self-motion through the environment in the anterior–posterior direction (Lestienne et al. 1977; Wei et al. 2010; Holten et al. 2013), we expected to find the least sway to be induced by the lowest (2°/s) speed and increasing sway magnitudes with increasing translational dot speeds. In addition to examining the postural sway magnitude as a function of different dot speeds, we determined whether a directional anisotropy in medial–lateral sway existed for these stimulus speeds. Based on the results of other studies (e.g., Ehrenfried et al. 2003; Ravaioli et al. 2005; Tsutsumi et al. 2010), we expected this not to be the case.

### Methods

#### Observers

Twelve healthy observers participated in the experiment. All had normal or corrected-to-normal visual acuity and were naïve to the purpose of the study. The experiment did not utilize any invasive techniques, substance administration or psychological manipulations. Therefore, compliant with Dutch law, this study only required and received approval from our internal faculty board (Faculty’s Advisory Committee under the Medical Research Human Subjects Act, WMO Advisory Committee) at Utrecht University. Written informed consent was obtained from all observers. The experiment was conducted according to the principles expressed in the Declaration of Helsinki. By signing the informed consent, observers indicated to have read and agreed with both the rules regarding participation and proper (laboratory) behavior, and the researchers’ commitments and privacy policy. Observers were also informed that they could stop participating in the experiment at any time and that all data would be analyzed anonymously.

#### Stimuli and apparatus

Stimuli were generated on a MacPro and projected on a flat rear projection screen by a DepthQ HDs3D-1 projector (refresh rate 120 Hz, resolution 1280 × 720). Postural sway of the observers was measured using a custom-made forceplate (ForceLink BV) with a sample rate of 1000 Hz. Stimuli (87° horizontal × 56° vertical, 220 × 124.5 cm) were viewed from a distance of 116 cm. The stimuli were composed of 5000 randomly placed dots (diameter ~0.13°) with an unlimited lifetime. The dot density was 0.91 dots/deg^2^. Dots could translate either leftward or rightward with a constant angular speed of 2°/s, 4°/s, 8°/s, 16°/s, 32°/s or 64°/s. Dots reaching the edge of the screen were randomly replaced at the other side of the screen. A fixation point (diameter ~1°) was presented at the center of the screen to prevent pursuit eye movements that occur when observers track the translating dots of the stimulus. We wanted to avoid eye movements since they are known to affect postural sway (Glasauer et al. 2005). The fixation point occluded some dots of the translating stimulus.

#### Procedure

Observers stood in a completely darkened room on a forceplate that was covered with foam. They did not wear goggles to limit the field of view, since no disturbing lights or objects could be viewed in the periphery. Observers were instructed to place their feet in a semi-tandem position (i.e., toes of one foot are level with the inside arch of the other foot) and position them a few centimeters apart so that the feet and the knees did not touch each other. Observers were asked to keep their weight equally distributed between their feet and hold their arms at their sides. The experiment started as soon as observers indicated that they were ready to start. The translating stimulus was presented for 4 s, and observers were instructed to fixate on the fixation point. Eye movements were not recorded. In total, 12 conditions (i.e., two motion directions: left and right; six dot speeds: 2°/s, 4°/s, 8°/s, 16°/s, 32°/s and 64°/s) were presented 18 times to observers during the complete experiment. The translating stimuli were interleaved by dynamic noise stimuli with a random duration between 3.5 and 4 s. The dynamic noise stimulus was similar to the translating stimulus, except that the dots were randomly replaced every frame. To prevent observers from being actively aware of their posture, they had to perform a memory task. During the presentation of the dynamic noise stimulus, the fixation point contained either a red, green, blue or yellow color. The task of observers was to count how often a particular dot color was presented and report it at the end of each block. Observers first performed three blocks of 24 trials containing six trials per condition in a random order. In these blocks, the visual stimulus translated either leftward or rightward and contained a constant angular speed of 4°/s or 32°/s. Subsequently, six blocks of 24 trials containing a visual stimulus that translated either leftward or rightward with a speed of 2°/s, 8°/s, 16°/s or 64°/s were presented to observers. Each condition was presented three times in a random order in these blocks of trials. The total duration of each block was approximately 3 min. Observers were allowed to take a short break (~3 min) between blocks.

#### Analysis

After down-sampling the data from the forceplate to 125 Hz, the center of pressure (COP) in the medial–lateral direction was calculated. To remove measurement noise, the COP data were filtered with a zero-phase fourth-order Butterworth filter (cutoff frequency 10 Hz). To be able to calculate the COP deviation per trial, COP at stimulus onset served as baseline. The baseline was thus the first data point of each trial, which was set to zero. For the total duration of the stimulus and the minimal duration of dynamic noise (4 + 3.5 s), COP deviation from baseline was determined. For each condition, we checked for outliers in the COP deviation of all trials of that condition, which are possibly the result of excessive postural adjustments. Any trial belonging to a certain condition was discarded from analysis when the COP deviation fell outside the median ± 3 standard deviations of all trials of that condition. We used three standard deviations as a criterion since this was just sufficient to remove a trial in which an observer reported that he made a step to maintain his balance. Overall, between 4.6 % (10 trials) and 10.6 % (23 trials) with a median of 8.1 % (17.5 trials) were removed for different observers. The removed trials were distributed quasi-equally across conditions. For each observer, the COP deviation from baseline was averaged over up to 18 trials per condition (14 trials minimum). Analogous to Holten et al. (2013), the area under the curve between 1 and 4 s after stimulus onset was subsequently calculated for each condition and was used as a measure of sway magnitude. The area under the curve of the first second after stimulus onset was analyzed separately for each condition.

#### Statistics

A repeated measures analysis of variance (ANOVA) was performed on the area under the curve to examine significant differences in postural sway between stimulus conditions. In case the assumption of sphericity was violated, the number of the degrees of freedom was adjusted using the Greenhouse–Geisser method. Partial eta squared (*η*_*p*_^2^) was used to report effect sizes for the main effects. Post hoc pairwise comparisons with a Sidak correction were used to examine significant differences between conditions.

### Results and discussion

**Fig. 1:**
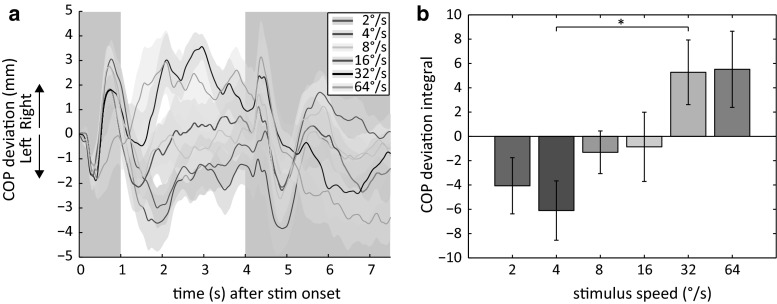
Center of pressure (COP) deviation from baseline (**a**) and the COP deviation integral (**b**) averaged across observers for different dot speeds. **a** The COP trajectories of both motion directions are collapsed with all trials converted to a rightward translating stimulus. Positive COP values represent rightward sway, which is in the same direction as the motion of the stimulus. Negative COP values represent leftward sway, which is in the opposite direction as the stimulus motion. The *colored areas* represent the standard error of the mean and the *bold lines* the mean across observers of a single condition. Dynamic noise was presented 4 s after stimulus onset. COP trajectories in the *gray regions* represent the postural sway in the first second after stimulus onset and during the presentation of dynamic noise. **b** The COP deviation integral is the area under the *curve* between 1 and 4 s after stimulus onset (**a**). A positive integral represents rightward sway and a negative integral leftward sway. *Error bars* represent the standard error of the mean. The *dash* indicates a significant difference between conditions (color figure online)

For each dot speed, the COP trajectory averaged across observers is shown in Fig. 1a. The COP trajectories of leftward and rightward translation are collapsed since there is no significant difference in the postural sway magnitude (represented by the area under the curve) between these conditions [paired-samples *t* test: *t*(11) = .755, *p* = .466, *r* = .22]. This resulted in up to 36 trials per dot speed. All trials are converted to rightward translation of the dots. A repeated measures ANOVA was performed on all conditions with the factor dot speed (six levels: 2°/s, 4°/s, 8°/s, 16°/s, 32°/s and 64°/s). A main effect of the speed of the translating stimulus on the postural sway magnitude was observed [*F*(5,55) = 6.18, *p* < .001, *η*_*p*_^2^ = .36]. Post hoc pairwise comparisons revealed that the magnitude of postural sway induced by a stimulus speed of 4°/s (−6.105) differed from the postural sway magnitude generated by a translating stimulus with a speed of 32°/s (5.272; see Fig. 1b).

The observed difference in postural sway between stimulus speeds is not the result of more postural sway caused by higher stimulus speeds per se. A separate repeated measures ANOVA performed on the *absolute* area under the curve showed no main effect of stimulus speed on the postural sway magnitude [*F*(5,55) = 1.24, *p* = .303, *η*_*p*_^2^ = .10]. Hence, as is apparent from Fig. 1, the observed postural sway differences are the result of postural sway in *different directions* for different dot speeds.

As is apparent from Fig. 1a, directly after stimulus onset (0–0.5 s) observers sway in a direction opposite to the stimulus motion. This initial movement is followed by a COP displacement in the opposite direction (0.5–0.75 s). For most stimulus speeds, this COP displacement is again followed by a movement of the observers in a direction opposite to the stimulus motion. At approximately one second after stimulus onset, the COP deviation has returned to baseline. Considering the time period between 0 and 1 s after stimulus onset, there is no main effect of the stimulus speed on the area under the curve [*F*(2.99,32.85) = 1.64, *p* = .200, *η*_*p*_^2^ = .13]. This indicates that the postural sway within the first second after stimulus onset is consistent across stimulus speeds. In other words, the difference in postural sway across stimulus speeds occurs after the first second of sway.

The results of the first experiment are not in line with our expectation that higher dot speeds would induce more medial–lateral postural sway. A different interpretation of the visual stimulus at different dot speeds could serve as a possible explanation of the current results. It has been shown that the direction of medial–lateral sway is generally in the same direction as the stimulus motion when observers directly fixate the motion (Bronstein and Buckwell 1997; Guerraz et al. 2001; Meyer et al. 2013). When observers fixate a real object in front of the motion pattern, the postural sway is in the opposite direction (Bronstein and Buckwell 1997; Guerraz et al. 2001). However, fixating a virtual object in front of the stimulus does not appear to elicit significant postural sway at all (Meyer et al. 2013). Perhaps at low dot speeds, observers interpreted the fixation point as translating on top of a stationary background, while at faster dot speeds the fixation point was perceived to be stationary and the background as moving. If this is the case, this different interpretation of the stimulus at different dot speeds may result in sway in opposite directions. In addition, motion parallax has been shown to influence the postural sway direction as well (Bronstein and Buckwell 1997; Guerraz et al. 2001). If the fixation point is perceived in front of the visual stimulus at low speeds and at the same depth plane at higher speeds, this may also result in postural sway in opposite directions. However, this is not very likely since the presentation of the fixation point and the dots are coplanar.

One could argue that the retinal motion induced by a slow-velocity stimulus is indistinguishable from the retinal motion occurring during self-generated sway (natural sway velocity), as these velocities are rather similar. However, this would not explain the difference in postural sway direction across stimulus speeds. Namely, when observers perceive, for example, the retinal motion induced by a rightward moving stimulus as induced by leftward self-generated sway, they will compensate by swaying to the right. The results of our study show that this is not the case for low stimulus speeds.

In order to get a handle on the effect of the (perceived) depth ordering, varying the fixation point contrast independent of stimulus contrast might suffice. In daily life, aerial perspective cues cause objects that are further away from the observer to have a lower contrast than objects that are closer to the observer (O’Shea et al. 1993). Presenting two stimuli with a different luminance on the same background cause the higher-contrast stimulus to appear closer than the lower-contrast stimulus (O’Shea et al. 1993). Varying the fixation point contrast might therefore change the perceived depth ordering of the fixation point and the translating dots, respectively, and therefore also the direction of induced postural sway. A fixation point that is of higher contrast than the translating dots (i.e., brighter than the dots on a dark background) can be perceived in front of the translating dots and therefore cause no sway or sway opposite to the motion direction of the stimulus. A fixation point with a lower contrast (i.e., darker than the dots on a dark background) may be perceived behind or at the same depth plane as the translating dots and may induce sway in the same direction as the motion direction of the stimulus.

## Experiment 2

In Experiment 2, we varied the fixation point contrast to determine whether it could alter the direction of postural sway induced by a linear translating stimulus. Moreover, the contrast of the translating dots was manipulated independently to investigate whether the perceptual strength of a visual stimulus influences the postural sway magnitude. For all dot speeds used, we expected less postural sway with decreasing dot contrast, since this stimulus is probably a perceptually ‘weaker’ stimulus than its high-contrast counterpart. So, in the current experiment, we varied the contrast of the translating dots, the contrast of the fixation point and the angular speed of the translating dots in a factorial design.

### Methods

The methods of Experiment 2 are identical to Experiment 1, except for the differences mentioned below.

#### Observers and stimuli

A new group of 20 healthy observers participated in the experiment. The stimuli subtended 85° by 55° and the dots of the translating stimulus could translate either leftward or rightward with a constant angular speed of 4°/s, 16°/s or 32°/s. We selected these speeds since they generated almost no consistent sway (16°/s) or sway in opposite directions (4°/s, 32°/s) in the first experiment. The dot density was 0.93 dots/deg^2^. The fixation point and the dots composing the linear translating stimulus were presented on a dark background (0.045 cd/m^2^), and either contained a Weber contrast of approximately 3 % (0.17 cd/m^2^), 9 % (0.45 cd/m^2^) or 92 % (4.2 cd/m^2^).

#### Procedure

In total, 54 conditions (i.e., two motion directions: left and right; three dot speeds: 4°/s, 16°/s and 32°/s; three contrast levels of fixation point: 3 %, 9 % and 92 %; three contrast levels of dots of the translating stimulus: 3 %, 9 % and 92 %) were presented 10 times to observers during the experiment. Each block contained all 54 conditions, and each condition was presented once per block in a random order. A block therefore contained 54 trials, each lasting between 7.5 and 8 s (translation + dynamic noise). The total duration of a block was approximately 7 min. In total, observers had to perform 10 blocks during the complete experiment.

The last nine of the 20 observers who participated in this experiment performed an extra block of trials after finishing the main experiment. This block was performed specifically to examine whether the postural sway direction changed when the fixation point was perceived as foreground or as background. The 40 trials of this block all contained a dot speed of 4°/s, a dot contrast of 92 % and a fixation point contrast of 9 %. A fixation point contrast of 9 % was used to be able to present the dots on top of the fixation point, which was the case in half of the trials. Although presented in the same 2D depth plane, we assumed that observers would perceive the dots in front of the fixation point since the dots were not occluded, but did occlude the fixation point. In the other trials, the fixation point occluded the dots, as was the case in the main experiment. We assumed that in this condition the fixation point would be perceived as foreground and the dots as background. The dots translated either to the left or to the right. The translation direction and the occlusion of the translating dots by the fixation point were counterbalanced. The task of observers was the same as the task during the preceding blocks of trials (count how often a fixation point color was presented). At the end of the experiment, these observers were debriefed about their visual experience of the translating pattern in each condition.

#### Analysis

As in Experiment 1, we checked for outliers in the COP deviation (COP deviation fell outside the median ± 3 standard deviations of all trials of that condition) that are possibly the result of excessive postural adjustments. Overall, between 0.18 % (one trial) and 5.9 % (32 trials) with a median of 2.1 % (11.5 trials) were removed for different observers. The removed trials were distributed quasi-equally across conditions. For each observer, the COP deviation from baseline was averaged over up to 10 trials per condition (eight trials minimum).

The extra block of trials was analyzed separately but in a similar fashion compared with the analysis of the main experiment and Experiment 1. Between 0 % (0 trials) and 10 % (4 trials) with a median of 2.5 % (1 trial) of the trials were removed for different observers, since the COP trajectories of these trials fell outside the median ± 3 standard deviations of all trials of that condition.

#### Statistics

Several repeated measures analyses of variance (ANOVA) were performed on the area under the curve to examine significant differences in postural sway between stimulus conditions. In case the assumption of sphericity was violated, the number of the degrees of freedom was adjusted using the Greenhouse–Geisser method. Partial eta squared (*η*_*p*_^2^) was used to report effect sizes for main and interaction effects. Post hoc pairwise comparisons with a Sidak correction were used to examine significant differences between conditions.

### Results

#### All conditions

**Fig. 2:**
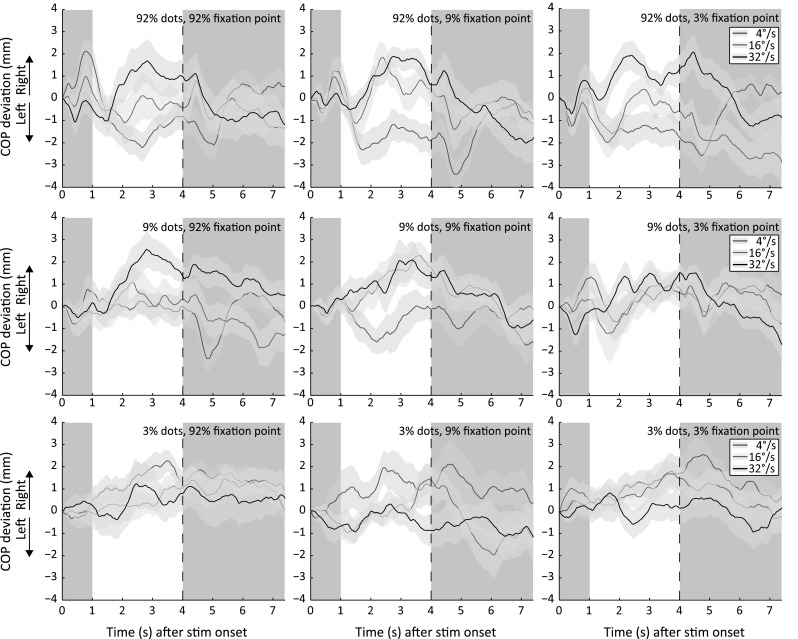
Center of pressure (COP) deviation from baseline averaged across observers for different dot contrasts, fixation point contrasts and dot speeds. Each *row* of *panels* represents a different dot contrast and each *column* a different fixation point contrast. For formatting details, see Fig. 1 (color figure online)

For each condition, the COP deviation from baseline after stimulus onset is displayed in Fig. 2. Each row (three horizontal panels) in the figure represents a different contrast of the translating dots and each column (three vertical panels) a different fixation point contrast. The COP trajectories of leftward and rightward translation are collapsed since no difference in the area under the curve between these motion directions was observed [paired-samples *t* test: *t*(19) = −.028, *p* = .978, *r* = .006]. This resulted in up to 20 trials per condition. All trials are converted to rightward translation of the dots.

**Fig. 3:**
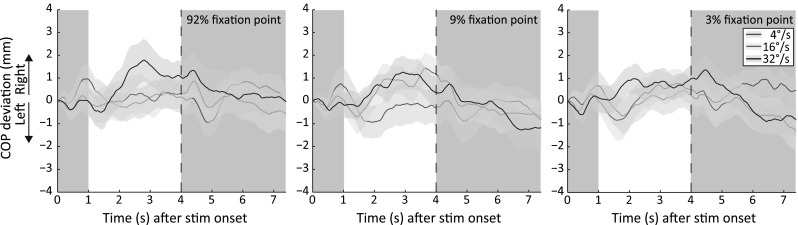
Center of pressure (COP) deviation from baseline collapsed over the three dot contrasts and averaged across observers for different fixation point contrasts and dot speeds. For formatting details, see Fig. 1. Each *panel* depicts the COP trajectories of each fixation point contrast when the COP trajectories of all dot contrasts are collapsed. For example, the *left panel* depicts the averaged COP trajectories of the *left panels* in Fig. 2 (color figure online)

A repeated measures ANOVA was performed on all conditions with the factors dot speed (three levels: 4°/s, 16°/s and 32°/s), contrast of fixation point (three levels: 3, 9 and 92 %) and the contrast of the translating dots (three levels: 3, 9 and 92 %). A main effect of the contrast of the translating dots (i.e., dot contrast) on the sway magnitude was observed [*F*(2,38) = 3.25, *p* = .0497, *η*_*p*_^2^ = .146], with on average a lower sway magnitude for higher-contrast translating dots. This finding is probably not the result of less postural sway caused by high-contrast dots but rather by the finding that the postural sway is in different directions for different dot speeds. The postural sway of these speeds (4°/s and 32°/s) cancels each other when averaged. As a consequence, almost no net postural sway is observed for the high-contrast dots. We performed a separate 3 × 3 repeated measures ANOVA on the absolute area under the curve to examine whether high-contrast stimuli induce more postural sway than low-contrast stimuli. The results showed that this is not the case [*F*(2,38) = 3.25, *p* = 0.748, *η*_*p*_^2^ = .015]. For both analyses, post hoc pairwise comparisons did not show a significant difference between any of the dot contrasts. No main effect was observed for dot speed [*F*(1.51,28.7) = 2.18, *p* = .127, *η*_*p*_^2^ = .103] and the fixation point contrast [*F*(2,38) = .074, *p* = .929, *η*_*p*_^2^ = .004]. When the COP trajectories of the three dot contrasts are collapsed, it can be seen that the fixation point contrast does not affect the postural sway magnitude (Fig. 3).

We found a significant interaction between dot speed and dot contrast of the translating stimulus [*F*(4,76) = 12.83, *p* < .001, *η*_*p*_^2^ = .40]. This interaction is probably caused by the lowest (4°/s) and highest (32°/s) dot speed used in this experiment. The postural sway induced by a translating pattern containing a dot contrast of 92 % and a dot speed of 4°/s (Fig. 2, blue line, top panels) is in the opposite direction compared with the postural sway induced by a translating pattern containing the same speed but with a contrast of 3 % (Fig. 2, blue line, bottom panels). The similar sway pattern can be observed for dots translating at 32°/s (Fig. 2, black line, same panels), but the effect is in the opposite direction compared with that is observed for the 4°/s moving dots. No significant interactions were found between fixation point contrast and dot contrast [*F*(4,76) = .50, *p* = .736, *η*_*p*_^2^ = .03], fixation point contrast and dot speed [*F*(2.18,41.48) = .61, *p* = .657, *η*_*p*_^2^ = .03] and fixation point contrast, dot contrast and dot speed [*F*(8,152) = .99, *p* = .449, *η*_*p*_^2^ = .05] of the translating stimulus.

#### Fixation point contrast collapsed

**Fig. 4:**
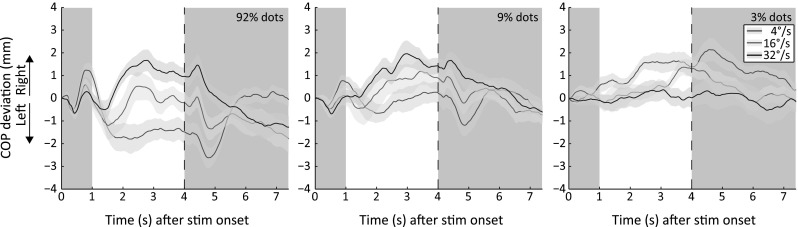
Center of pressure (COP) deviation from baseline collapsed over the three fixation dot contrasts and averaged across observers for different dot contrasts and dot speeds. For formatting details, see Fig. 1. Each *panel* represents the COP trajectories of each dot contrast when the COP trajectories of all fixation point contrasts are collapsed. For example, the COP trajectories of the *left panel* are the averaged COP trajectories of the *upper panels* in Fig. 2 (color figure online)

Since we did not observe a main effect or any interaction regarding the fixation point contrast, we collapsed the COP trajectories of the three fixation point contrasts (Fig. 4). We performed separate one-way ANOVAs for each dot contrast and dot speed to examine the observed interaction between the dot speed and the dot contrast of a translating pattern to a larger extent. For a dot contrast of 92 %, we found a main effect of dot speed [*F*(2,38) = 12.24, *p* < .001, *η*_*p*_^2^ = .392] on the postural sway magnitude. Post hoc pairwise comparisons showed that for this dot contrast (92 %), a speed of 4°/s (area under the curve: −3.957) induced postural sway in a direction opposite to the sway induced by a translating pattern with a dot speed of 32°/s (area under the curve: 2.556, *p* < .001). Furthermore, the pattern translating at 4°/s induced more postural sway than the pattern translating at 16°/s (area under the curve: −.629, *p* = .023). One-sample *t* tests with α adjusted to .0167 to correct for multiple comparisons were used to determine whether the postural sway magnitude significantly differed from zero. The results showed that significant sway was generated by the lowest (*p* = .010) and highest (*p* = .015) dot speed. For a dot contrast of 9 %, the dot speed did not influence the postural sway magnitude [*F*(1.27,24.09) = 2.60, *p* = .113, *η*_*p*_^2^ = .120]. One-sample *t* tests with α adjusted to .0167 to correct for multiple comparisons showed that significant postural sway was only generated by the highest dot speed (*p* = .005). For the lowest (3 %) dot contrast, an effect of dot speed on the postural sway magnitude was again observed [*F*(2,38) = 4.83, *p* = .014, *η*_*p*_^2^ = .20]. Post hoc pairwise comparisons showed that the pattern translating at 4°/s (area under the curve: 3.650) induced more postural sway than the pattern translating at 32°/s (area under the curve: .215, *p* = .033). One-sample *t* tests with α adjusted to .0167 to correct for multiple comparisons showed that significant postural sway was only generated by the lowest dot speed (*p* < .001).

We also examined the effect of manipulating dot contrast of a single dot speed on the postural sway magnitude. The postural sway direction of the pattern translating at 4°/s changes with the dot contrast of the translating pattern [*F*(2,38) = 16.07, *p* < .001, *η*_*p*_^2^ = .46]. The postural sway direction that is induced by a translating pattern containing a dot contrast of 92 % and a dot speed of 4°/s is opposite (area under the curve: −3.956) to the motion direction of the pattern. Post hoc pairwise comparisons showed that the postural sway caused by this condition differs in magnitude from the sway caused by a speed of 4°/s and a dot contrast level of 9 % (−0.223, *p* = .019). A 4°/s translating pattern with a dot contrast of 3 % induces postural sway (area under the curve: 3.65) in the same direction as the motion direction of the pattern. The sway caused by this condition also differs in magnitude from the sway caused by a translating pattern containing the same speed but a dot contrast of 92 % (*p* = .020) or 9 % (*p* < .001). The postural sway magnitude induced by a pattern translating at 32°/s is also influenced by the dot contrast [*F*(2,38) = 3.65, *p* = .036, *η*_*p*_^2^ = .16]. More postural sway is generated by a 32°/s translating pattern containing a high (92 %; area under the curve: 2.556) contrast than a low (3 %; area under the curve: 0.215) contrast (*p* = .035). No difference in postural sway is observed for each dot contrast when the pattern translated with a speed of 16°/s [*F*(2,38) = 1.34, *p* = .274, *η*_*p*_^2^ = .066]. All in all, the interaction between the dot speed and the dot contrast level of a translating pattern appears mainly to be induced by the patterns translating at a speed of 4°/s and 32°/s.

#### Effect of dots either in front of or behind fixation point

**Fig. 5:**
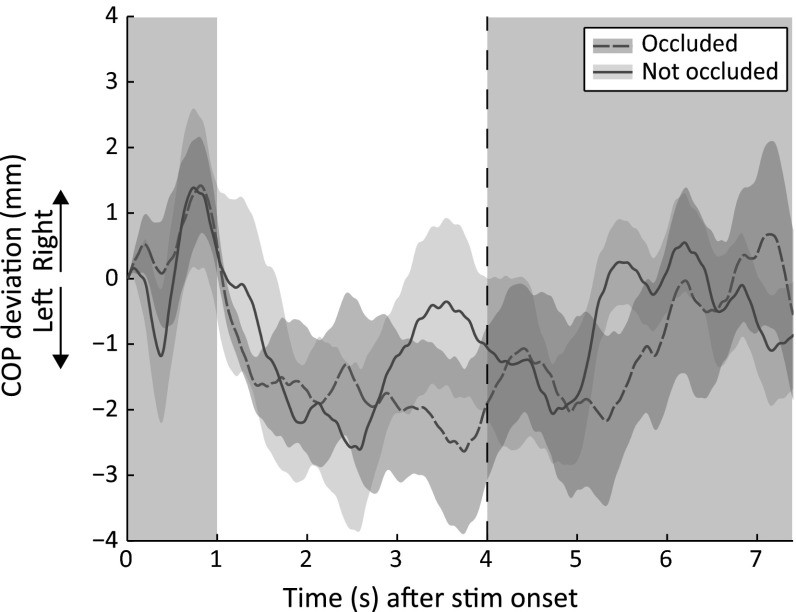
Center of pressure (COP) deviation from baseline averaged across nine observers for a pattern containing a dot contrast of 92 %, a fixation point contrast of 9 % and a dot speed of 4°/s. The *dashed line* represents the COP trajectory induced by a translating dot pattern when the translating dots were occluded by the fixation point. The *solid line* represents the COP trajectory induced by the translating dot pattern when the dots were not occluded by the fixation point. For formatting details, see Fig. 1 (color figure online)

The COP trajectories of the condition where the dots of the translating pattern were either occluded or not occluded by the fixation point are depicted in Fig. 5. A paired-samples *t* test between the area under the curve of both conditions did not show a significant difference in postural sway between the two conditions [*t*(8) = .347, *p* = .737, *r* = .12]. Debriefing revealed that seven of the nine observers perceived the fixation point at the foreground and the dots at the background in all conditions. Two observers did not remember whether they perceived the fixation point at the foreground or at the background, but they mentioned that they did not perceive any differences between conditions. The COP trajectories of these observers did not differ from the COP trajectories of the other observers.

## General discussion

We addressed the question whether manipulating the dot contrast and speed of a linear translating pattern would affect the postural sway magnitude. We assumed that a high-contrast stimulus is perceptually stronger and as a result induces more postural sway than a low-contrast stimulus. Furthermore, we examined whether manipulating the speed of the translating pattern affected the postural sway magnitude and investigated whether manipulating the fixation point contrast influenced the direction of postural sway.

### Fixation point contrast

Manipulating the fixation point contrast did not alter the postural sway magnitude. The most parsimonious explanation for this result is that most observers probably perceived the fixation point as being in front of the moving pattern in all conditions, irrespective of its contrast. We debriefed nine observers of which seven reported that this was the case and two observers did not remember whether they perceived the fixation point in front of or behind the moving pattern. This even holds for the added control trials. Even when the fixation point, which contained a similar contrast in both conditions, was partially occluded by translating dots, seven out of nine observers (two could not remember) still perceived the fixation point in front of the dots. Since the observers were able to make a judgment about the fixation point being in front of behind the dots, it is unlikely that they could not distinguish whether the fixation point was in the foreground or background with respect to the moving dots. However, it is possible that the observers were influenced by the preceding trials of the main experiment, where the fixation point always occluded the dots.

### Effect of dot contrast and dot speed

We did not observe a linear increase in postural sway magnitude with increasing dot contrast or dot speed, which is not in line with our initial expectations. However, manipulating the dot contrast and dot speed does affect the postural sway magnitude, since we observed an interaction between dot contrast and dot speed. This interaction is mainly caused by the lowest (4°/s) and highest (32°/s) dot speed, since a dot speed of 16°/s induces a postural sway magnitude that is in between the sway magnitudes of the lowest and highest dot speed in most conditions.

The finding that on average relatively low and relatively high speeds induce more directionally specific sway than intermediate speeds can possibly be explained by the existence of two independent visual motion sensor populations in visual cortex. One population tuned to low speeds and the other population tuned to a higher but overlapping speed range. Previous studies have provided evidence for the existence of two rather independent speed-tuned visual motion channels using transparent motion patterns (e.g., Verstraten et al. 1996, 1998, 1999; van der Smagt et al. 1999). Further evidence for the existence of two independent speed-tuned motion channels was provided by studies using binocular rivalry (van de Grind et al. 2001) and using visual evoked potentials to motion onset (Heinrich et al. 2004; Lorteije et al. 2008). The finding that in our study, an intermediate speed (16°/s) induces less sway than relatively high or low speeds, even in the high-contrast condition, might be the result of the two-motion channels canceling each other. If so, the previously described perceptual independence between these motion-processing channels does not hold for the observed postural sway. That is, if the two-motion-channel proposition can indeed serve as explanation, these do interact (i.e., lose independence) at this behavioral level.

Our previous study investigating anterior–posterior postural sway, generated by a single-speed optic flow stimulus (Holten et al. 2013), did show more postural sway for an optic flow pattern with an approximately similar speed (12°/s) as in the current study than for lower dot speeds. Moreover, relatively low speeds generated postural sway in the same direction as relatively fast speeds. However, the stimulus in this previous study differed (e.g., different dot density, motion direction, fixation point size) from the current study, and this may have caused the different findings between our previous study and the current study.

A remarkable finding of the current study is that the postural sway direction of the lowest speed (4°/s) changes with dot contrast. Within the visual motion domain, integration has been shown to be facilitated when the stimulus contains more noise (Lorenceau 1996), or for lower-contrast stimuli, where for instance surround suppression weakens or even becomes facilitation (e.g., Tadin et al. 2003, 2008; Pack et al. 2005). Analogous to these findings, it is possible that at a high dot contrast, *suppression* occurs at a response or behavioral level. This suppression may become weaker when the dot contrast decreases and even changes to facilitation or integration at the lowest dot contrast, hence the reversal in sway direction. Differences in suppression and integration may influence the internal representation of the visual stimulus and as a consequence influence the postural sway direction. For the highest stimulus speed, we did not observe a change in postural sway direction with decreasing stimulus contrast. Perhaps, the suppression is weaker for relatively high-speed than relatively low-speed stimuli. It is of interest that in recent studies on the influence of stimulus contrast in motion vision, such a reversal of effects did also disappear for higher-speed stimuli (Thompson et al. 2006; van der Smagt et al. 2010). Note, however, that the above serves mainly as an interesting analogue to, not necessarily as explanation for, the observed reversal of sway direction.

Manipulating the dot contrast influenced postural sway differentially for different speeds. For the lowest speed, dot contrast even determined the *direction* of postural sway. This finding and the finding that most observers indicated this was not accompanied by a change in visual experience (in that a different depth ordering was not perceived) clearly show that the observed effect of contrast cannot be explained by a differentially perceived depth ordering. If observers would have perceived a different depth ordering, it could have been an explanation for the observed opposite sway directions (Tanaka and Saito 1989; Bronstein and Buckwell 1997; Guerraz et al. 2001; Meyer et al. 2013). Rather, the relationship between the perceived strength of a visual stimulus and the resulting behavioral outcome (i.e., postural sway) appears not to be a straightforward (direct) stimulus–response relationship. Hence, it is tempting to assume that not the actual presentation of visual motion but an internal representation of the visual stimulus influenced postural sway.

In this light, it is noteworthy to discuss a previous study (Holten et al. 2014) that used an approximately similar speed (3°/s) and observed postural sway in the same direction as the stimulus motion. Based on our results at hand, one would have expected sway in a direction opposite to the stimulus motion using a stimulus speed of 3°/s. Whereas most observers also reported that they had the feeling that the fixation point was moving on a stationary background in our previous study, this sensation was not reported by the observers in the current study. It is conceivable that this difference in visual experience of the stimulus may explain the observed change in postural sway direction (Bronstein 1986; Guerraz et al. 2001).

One might argue that the memory task used in the experiments could have influenced our results since some studies have shown that performing a cognitive task influences postural sway (e.g., Pellecchia 2003; Riley et al. 2003, 2005). If our memory task indeed influenced postural sway, it is still unlikely that it affected our overall results, since the memory task was performed during the presentation of random noise that interleaved the translating stimuli. Moreover, for all stimulus conditions, the task was similar. In addition, in Experiment 1 all conditions were randomly presented within a block of trials. So, if cognitive load had influenced postural sway differentially at the beginning or at the end of a block of trials, this effect would have been canceled out between blocks. In Experiment 2, all conditions were randomly presented within a block of trials, but the order did not vary between blocks. However, each observer received a different trial order. The sway induced by the conditions that were present in both experiments is almost identical, indicating that it does not matter whether the conditions are randomized between blocks or not. The randomization of the stimuli across blocks of trials also decreases the effect on the average postural sway when observers did not return to the true baseline after the presentation of translating dots and the subsequent random noise.

## Conclusion

This study showed that the direction of postural sway changes with stimulus speed and contrast. However, the latter only holds for low speeds (i.e., 4°/s). We argue that this result can be explained by a different internal representation of the stimulus at different contrast levels. The fixation point contrast did not influence postural sway since it probably generated the same visual experience, namely that of moving dots behind a fixation point. All in all, the current study showed that the effect of stimulus contrast on postural sway depends on stimulus speed, suggesting that interactions between the visual, vestibular and proprioceptive system are influenced by the internal representation of a visual stimulus, rather than being driven by external stimulus characteristics only.

## Footnotes

1 Note that perceptual strength is not synonymous to stimulus strength. We are agnostic as to what causes the difference in perceptual strength. It can be either a difference in sensitivity or conspicuity between stimuli or a bias toward one of the stimuli.
